# Methylation-mediated BMPER expression in fibroblast activation *in vitro* and lung fibrosis in mice *in vivo*

**DOI:** 10.1038/srep14910

**Published:** 2015-10-07

**Authors:** Caijuan Huan, Ting Yang, Jiurong Liang, Ting Xie, Luis Cheng, Ningshan Liu, Adrianne Kurkciyan, Jessica Monterrosa Mena, Chen Wang, Huaping Dai, Paul W. Noble, Dianhua Jiang

**Affiliations:** 1 Department of Respiratory and Critical Care Medicine, Beijing Key Laboratory of Respiratory and Pulmonary Circulation Disorders, Beijing Chao-Yang Hospital-Beijing Institute of Respiratory Medicine, Capital Medical University, Beijing 100020, China; 2 Cedars-Sinai Medical Center, Department of Medicine, Los Angeles, CA 90048, USA; 3 China-Japan Friendship Hospital, Beijing, China

## Abstract

Idiopathic pulmonary fibrosis (IPF) is a progressive lung disease. Although the pathogenesis is poorly understood, evidence suggests that genetic and epigenetic alterations, such as DNA methylation, may play a key role. Bone morphogenetic proteins (BMPs) are members of the transforming growth factor-β (TGF-β) superfamily and are important regulators in IPF. Here we identified BMP endothelial cell precursor-derived regulator (BMPER) as a key regulator of fibroblast activation. BMPER is a secreted glycoprotein that binds directly to BMPs and may regulate TGF-β/BMP signaling, but its role in lung fibrosis is not clear. BMPER is highly expressed in human IPF lung fibroblasts compared to normal lung fibroblasts. Demethylation agent 5′-azacytidine decreased BMPER expression in fibroblasts, and attenuated the invasion and migration of IPF lung fibroblasts. Furthermore, siRNA-mediated reduction of BMPER in the human lung fibroblasts impaired cell migration and invasion. 5′-azacytidine treatment additionally regulated BMPER expression and reduced lung fibrosis in mice *in vivo*. These findings demonstrate that methylation of specific genes in fibroblasts may offer a new therapeutic strategy for IPF by modulating fibroblast activation.

Progressive lung fibrosis is an increasing cause of morbidity and mortality worldwide with limited therapeutic options. IPF, a particularly severe form of lung fibrosis, is a chronic, progressive, and often fatal interstitial lung disease of unknown etiology with an average survival of 2–3 years from diagnosis^1^,^2^,^3^. The current treatment options for IPF are very limited, therefore the development of novel treatment strategies that inhibited fibroblast activation, reduced epithelial apoptosis, or induced alveolar repaired is needed^3^,^4^,^5^. Epigenetic alterations can contribute to the pathogenesis of IPF^6^. Both DNA hypermethylation and hypomethylation have been observed in IPF^6^,^7^,^8^,^9^. Methylation of DNA occurs on cytosine residues that precede a guanosine in the DNA sequence (the CpG dinucleotide)^10^. Demethylating agent 5′-azacytidine normalized the phenotype of fibrotic fibroblasts *in vitro* and ameliorated experimental kidney fibrosis in mice^11^. Even though the effect of 5′-azacytidine is not limited to fibrosis, methylation of specific genes in fibroblasts is pivotal for perpetuating fibrogenesis^11^,^12^.

Both TGF-β and BMP signaling have been shown to play a role in lung fibrogenesis^13^. The balance between TGF-β and BMP signaling is crucial for lung homeostasis and fibrogenesis^13^,^14^. TGF-β signaling has been implicated to play an important role in fibrosis through stimulating matrix deposition^15^,^16^. In lung development, BMP signaling was shown to be essential for the growth and maintenance of lung epithelium^17^,^18^. BMP signaling plays a role in animal models of lung injury and in human lung disease^19^,^20^,^21^,^22^ by regulating fibroblast proliferation and transdifferentiation into myofibroblasts at the injury site^23^,^24^,^25^.

BMPER is a secreted glycoprotein that contains five cysteine-rich domains followed by a von Willebrand D domain and a trypsin-inhibitor domain^26^. BMPER is known to bind and modulate at least three BMPs (BMP-2, -4 and -6) and was originally identified in a screen for differentially expressed proteins in embryonic endothelial precursor cells^26^. High levels of BMPER antagonized BMP2-Smad5-Id1 signaling and prevented BMP2-mediated decrease of E-cadherin and hyperpermeability^27^. BMP activity controlled by BMPER regulates the proinflammatory phenotype of the endothelium^28^. BMPER can both promote and repress BMP signaling activity^29^. Mutations in the *BMPER* gene cause Diaphanospondylodysostosis in humans, possibly by disrupting the tight regulation of BMP signaling that governs development of the skeleton and other organs^30^, suggesting a critical role for BMPER in BMP signaling-mediated mesenchymal development. BMPER is highly expressed in malignant tumors and controls invasive cell behavior^31^. Our previous studies showed that increased invasion and migration capacities of fibroblasts contribute to severe lung fibrosis^32^,^33^. Inhibition of fibroblast migration can attenuate pulmonary fibrosis^34^. However, the role of BMPER in lung fibrosis has not been investigated. Interestingly, we found that the expression of several BMP binding proteins, including Gremlin1 and BMPER, was decreased in IPF fibroblasts after 5′-azacytidine treatment. The expression of Gremlin is increased in IPF lungs^35^. In this study, we tested the hypothesis that methylation-mediated expression of BMPER might be involved in lung fibrogenesis. We found that BMPER was up regulated in lung tissue and fibroblasts from IPF patients. Demethylation with 5′-azacytidine treatment resulted in decreased BMPER expression in primary lung fibroblasts, attenuated fibroblast activation *in vitro* and lung fibrosis *in vivo*. Furthermore, BMPER regulated lung fibrosis through TGF-β/BMP signaling.

## Results

### Up-Regulation of BMPER in IPF lung fibroblasts and tissues

**Figure 1:**
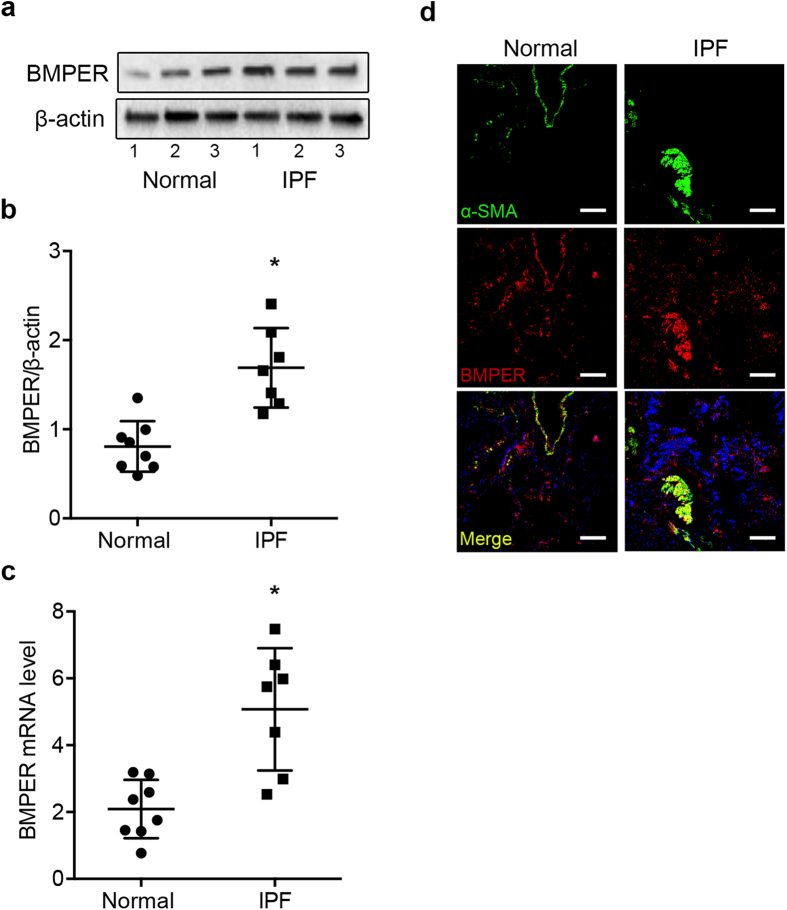
Increased BMPER expression in IPF lung fibroblasts and tissues. (**a**,**b**) BMPER protein levels were compared in primary lung fibroblasts derived from normal subjects and IPF patients with Western blot analysis (3 samples are shown). β-actin was used as a loading control. (**b**) Quantification of BMPER protein levels normalized to β-actin in normal and IPF lung fibroblasts by densitometry analysis (normal n = 8 and IPF n = 7; *P < *0.05,* data are mean and SEM, unpaired t test). (**c**) BMPER mRNA expression was compared in primary lung fibroblasts derived from normal subjects and IPF patients with qRT-PCR analysis (normal n = 8 and IPF n = 7, *P < 0.05, data are mean and SEM, unpaired t test). GAPDH was used as an internal control. (**d**) BMPER was co-localized with α-SMA in fibrotic foci. Representative confocal photomicrographs of immunofluorescence labeling for α-SMA (*green*), BMPER (*red*) and merged (*yellow*) images were from normal subjects and IPF patients. Scale bars, 20 μm. The experiments were repeated three times. The full size blots were shown in the Supplementary Fig. S1.

First we examined whether BMPER expression was aberrant between IPF and normal lung fibroblasts by Western blotting and qRT-PCR. Both the protein (Fig. 1a,b) and mRNA levels (Fig. 1c) of BMPER were elevated in IPF lung fibroblasts compared to normal lung fibroblasts. The increased BMPER protein levels were confirmed by immunofluorescence of human lung tissue. BMPER and α-SMA staining was increased in the fibrotic foci of IPF lung tissue (Fig. 1d). These findings indicate that BMPER levels are increased in IPF lung fibroblasts and lung tissues. It validates the need for further mechanistic insight into what role of BMPER may play in IPF.

### BMPER expression is decreased after 5′-azacytidine treatment in normal and IPF lung fibroblasts

**Figure 2:**
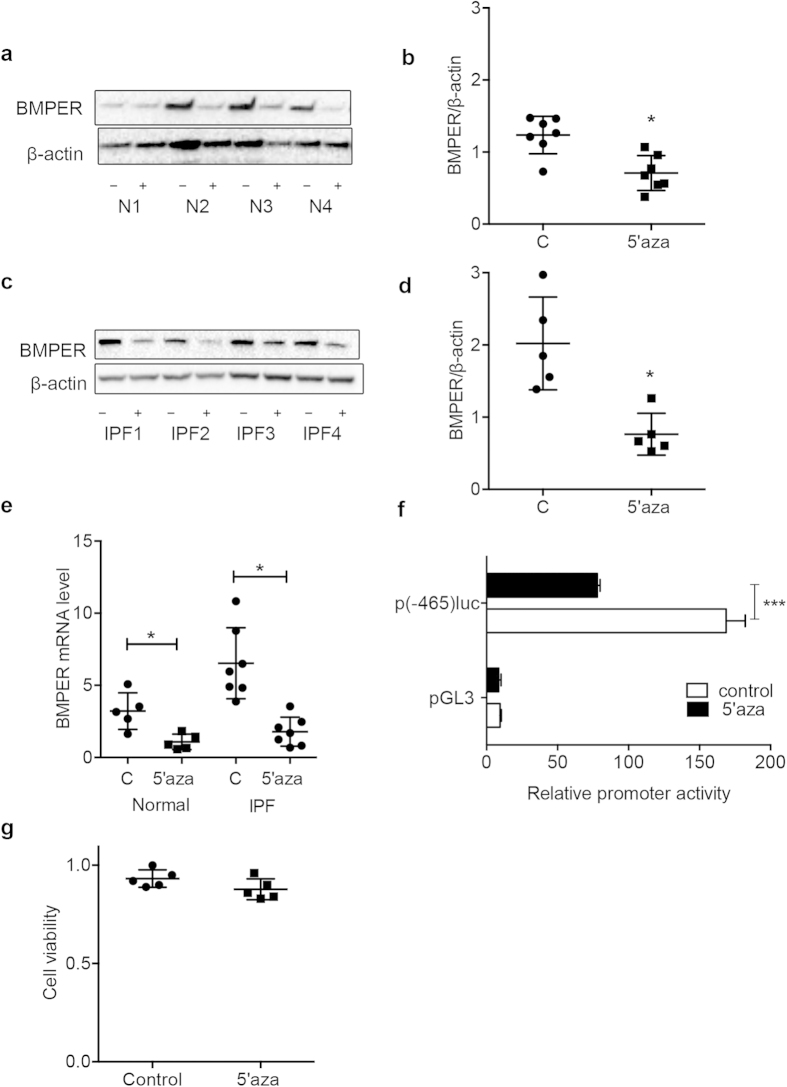
Demethylation decreased BMPER expression in normal and IPF fibroblasts and regulated the BMPER promoter activity. Primary normal (**a**,**b**) and IPF lung fibroblasts (**c**,**d**) were treated with 5′-azacytidine at 5 μM for 24 hours. BMPER protein levels in normal and IPF lung fibroblasts were analyzed with Western blotting (4 samples are shown). β-actin was used as a loading control. The relative amount was shown with densitometry analysis (normal n = 7 and IPF n = 5; N, normal; C, control; 5′aza, 5′-azacytidine; *P < 0.05; paired t test). (**e**) BMPER mRNA expression was assessed in human normal and IPF lung fibroblasts after 5 μM 5′-azacytidine treatment for 24 hours with qRT-PCR (normal n = 5 and IPF n = 7; C, control; 5′aza, 5′-azacytidine; *P < 0.05, paired t test). (**f**) Luciferase activity of human BMPER promoter was measured in HEK293 cells treated with 5′-azacytidine at 5 μM for 24 hours (***P < 0.001, data are mean and SEM, unpaired t test). (**g**) Cell viability of human lung fibroblasts with and without 5′-azacytidine treatment was measured with MTT assay. (n = 5, *P* > 0.05, paired t test). The full size blots were shown in the Supplementary Fig. S2.

DNA methylation has been suggested to play a role in fibrotic diseases^11^. In kidney, demethylating agent 5′-azacytidine ameliorated experimental renal fibrosis by mediating RASAL1 gene expression in mice *in vivo*^11^. In order to define the epigenetic regulation of lung fibrotic fibroblasts, a gene array was used to compare the gene expression changed in human lung fibroblasts from IPF patients after demethylation with 5′-azacytidine. It showed that after 5′-azacytidine treatment, the expression of several BMP binding proteins such as Gremlin1^35^, thrombospondin 1, SPARC-like 1, and BMPER were decreased compared to that of control fibroblasts. To directly investigate if elevated BMPER expression in IPF fibroblasts is associated with DNA methylation, we treated fibroblasts from IPF and normal lungs with the demethylation agent 5′-azacytidine. BMPER expression was decreased at both the protein and mRNA levels in the 5’-azacytidine treated normal and IPF fibroblasts (Fig. 2a–e).

Next we wanted to determine if the regulation of BMPER expression by demethylation was under the transcriptional level. A comparison of the 2000 base pairs upstream of the BMPER transcriptional start site in human identified positive regulatory *cis*-elements contained in the proximal promoter (−465 to +1)^36^. To determine possible regulation of BMPER by demethylation, we generated the BMPER promoter construct containing −465 to +1 to drive luciferase as a reporter. The BMPER promoter construct was transfected into HEK293 cells. The cells were treated with 5′-azacytidine 24 hours after transfection, and then harvested for luciferase activity measurement 48 hours after transfection. Consistent with a previous report^36^, we found that the proximal promoter (−465 to +1) contained an activating regulatory element (Fig. 2f). The promoter activity was significantly reduced after 5′-azacytidine treatment (Fig. 2f), indicating that BMPER expression is regulated by demethylation at the transcriptional level.

Additionally, cell viability was not significantly changed with 5′-azacytidine treatment as determined by MTT staining (Fig. 2g), suggesting that the decreased expression of BMPER by demethylation was not due to cytotoxic effects of 5′-azacytidine.

### 5′-azacytidine treatment diminishes the matrix production of IPF lung fibroblasts

**Figure 3:**
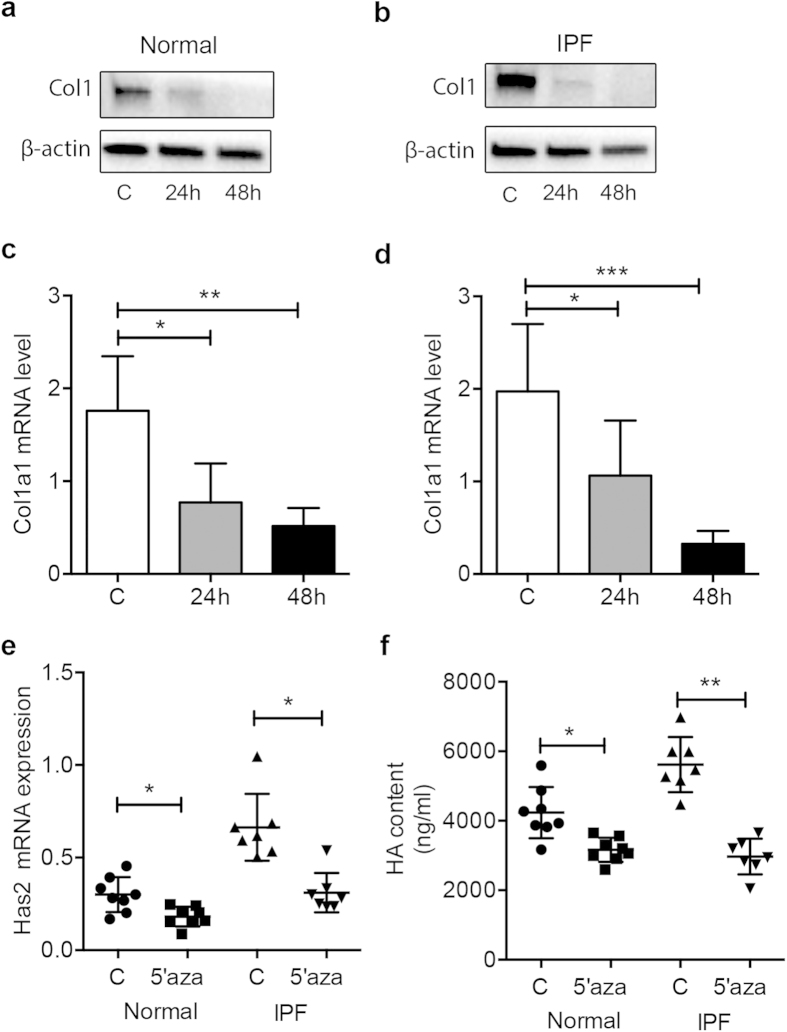
Demethylation correlated with the extracellular matrix production of primary lung fibroblasts. Extracellular matrix production of primary normal and IPF fibroblasts treated with 5′-azacytidine at 5 μM or control medium for 24 hours or 48 hours was assessed with Western, RT-PCR, and ELISA. Col1 protein levels in normal (**a**) and IPF (**b**) lung fibroblasts were analyzed with Western blotting. β-actin was used as a loading control (normal n = 3 and IPF n = 3). Col1 mRNA levels in normal (**c**) and IPF (**d**) lung fibroblasts were assessed with RT-PCR. GAPDH was used as a loading control. (Col1, Collagen 1, C, control, normal n = 4 and IPF n = 6, *P < 0.05; **P < 0.01; ***P < 0,001; data are mean and SEM, unpaired t test) (**e**). Has2 mRNA expression in primary normal and IPF fibroblasts treated with 5′-azacytidine were assessed with RT-PCR (C, control, normal n = 8 and IPF n = 7. *P < 0.05, paired t test). (**f**) The levels of HA in the supernatant of normal and IPF lung fibroblasts treated with 5′-azacytidine at 5 μM for 24 hours (C, control, HA, Hyaluronan, Normal n = 8 and IPF n = 7, *P < 0.05; **P < 0.01, paired t test). The full size blots were shown in the Supplementary Fig. S3.

IPF is a terminal illness characterized by unremitting matrix deposition in the lung^33^. These matrices include Collagen I (Col1), hyaluronan (HA) and hyaluronan synthase 2 (Has2)^5^,^37^. To gain insight into the role of BMPER in the progression of lung fibrosis, we next investigated if and how 5′-azacytidine treatment affects matrix production. After normal and IPF fibroblasts were treated with 5′-azacytidine, western blotting analysis showed that Col1 protein (Fig. 3a,b) and mRNA levels (Fig. 3c,d) were decreased in both normal and IPF lung fibroblasts. Also Has2 mRNA expression was decreased after 5′-azacytidine treatment (Fig. 3e). HA ELISA confirmed that after 5′-azacytidine treatment, the production of HA in the supernatant was decreased (Fig. 3f). Taken together, these data imply that down-regulated BMPER expression correlates with the decreased extracellular matrix production in lung fibroblasts.

### 5′-azacytidine treatment reduces the invasion and migration capacities of IPF fibroblasts

**Figure 4:**
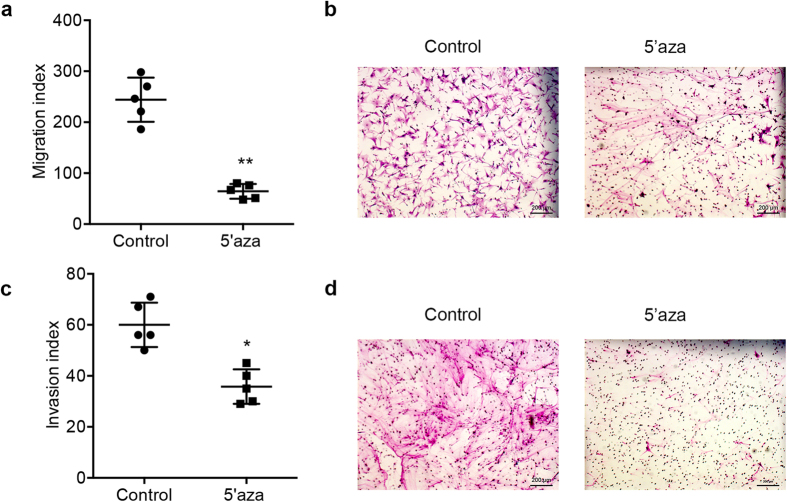
Reduced invasion and migration capacities of IPF fibroblasts treated with 5′-azacytidine. The invasion and migration capacities of primary IPF fibroblasts treated with 5′-azacytidine at 5 μM for 24 hours were assessed. (**a**) Migration capacity of IPF fibroblasts untreated and treated with 5′-azacytidine was assayed with a Boyden chamber (**P < 0.01, Data are mean and SEM, unpaired t test, n = 5 patients). (**b**) Representative images of migration from IPF fibroblasts untreated and treated with 5′-azacytidine (Scale bars, 200 μm). (**c**) Invasion capacity of IPF fibroblasts untreated and treated with 5′-azacytidine (*P < 0.05, Data are mean and SEM, unpaired t test, n = 5 patients). (**d**) Representative images of invasive IPF fibroblasts from IPF fibroblasts untreated and treated with 5′-azacytidine (Scale bars, 200 μm).

To investigate whether demethylation would affect IPF fibroblast behaviors, we measured the migration and invasion capacities of IPF fibroblasts after 5′-azacytidine treatment. Our results showed that after 5′-azacytidine treatment, both the migration (Fig. 4a,b) and invasion capacities (Fig. 4c,d) of the cells were decreased compared to untreated cells. These data suggest that demethylation may not only affect matrix production, but also change the fibroblast behavior.

### TGF-β/BMP signaling is regulated by BMPER

**Figure 5:**
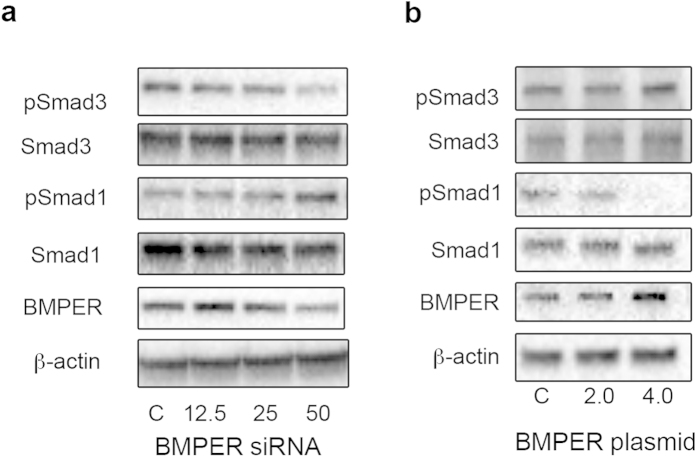
BMPER modulates fibroblast activation by facilitating TGF-β/BMP signaling. Western blot analysis was used to assess p-Smad3, Smad3, p-Smad1, Smad1 and BMPER expression levels in MRC5 cells (**a**) Cells were transfected with either 12.5–50 pmol BMPER siRNA or control siRNA 48 hours and (**b**) Cells were transfected with either 2–4 μg BMPER plasmid or control empty plasmid 48 hours. β-actin was used as a loading control. The experiments were repeated three times (C, control). The full size blots were shown in the Supplementary Fig. S4.

Both TGF-β and BMP signaling have been shown to play a role in lung fibrogenesis. The balance between TGF-β and BMP signaling is crucial for lung homeostasis and lung fibrosis^13^,^14^. In general, Smad2/3 transmit TGF-β signaling, while BMPs signal through Smad1/5/8^13^. We hypothesized that BMPER may mediate TGF-β/BMP signaling through Smad phosphorylation during demethylation. We therefore used siRNA interference (loss-of-function) and overexpression of BMPER (gain-of-function) in MRC5 cells to examine Smad-mediated TGF-β/BMP signaling that occurs downstream of BMPER. When BMPER was knocked down with BMPER specific siRNA, BMPER expression was reduced (Fig. 5a). The phosphorylation levels of Smad3 were attenuated, whereas the phosphorylation levels of Smad1 were increased (Fig. 5a). In contrast, the phosphorylation levels of Smad3 were increased and the phosphorylation levels of Smad1 were decreased in MRC5 cells with BMPER overexpression (Fig. 5b). These results show that BMPER can modulate TGF-β/BMP signaling, in which BMPER may activate TGF-β signaling and inhibit the BMP signaling pathway.

### BMPER regulates cell migration and invasion in fibroblasts

**Figure 6:**
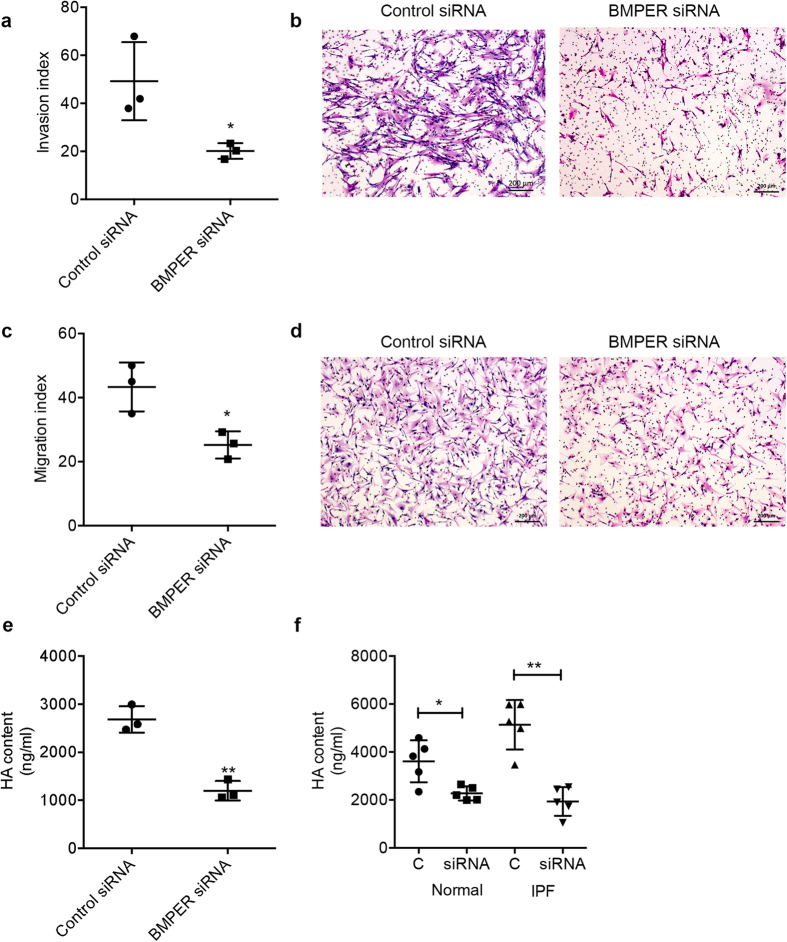
Knocking down BMPER with siRNA reduced migration and invasion capacities and matrix production of fibroblasts. (**a**) Cell invasion capacity of MRC5 cells transfected with BMPER siRNA or control siRNA (*P < 0.05, data are mean and SEM, unpaired t test, n = 3 times). (**b**) Representative images of Invasion (Scale bars, 200 μm). (**c**) Cell migration capacity of MRC5 cells transfected with BMPER siRNA or control siRNA was assayed with a Boyden chamber (*P < 0.05; data are mean and SEM, unpaired t test, n = 3 times). (**d**) Representative images of migration (Scale bars, 200 μm). (**e**) HA concentration of MRC5 cells transfected with BMPER siRNA or control siRNA (HA, Hyaluronan; **P < 0.01; data are mean and SEM, unpaired t test; n = 3 times). (**f**) HA concentration of normal and IPF lung fibroblasts transfected with BMPER siRNA or control siRNA (HA, Hyaluronan; *P < 0.05; **P < 0.01; data are mean and SEM, unpaired t test, normal n = 5 subjects and IPF n = 5 patients).

We next determined the functional role of BMPER in lung fibroblasts. Knowing that BMPER is a crucial regulator of TGF-β/BMP pathway activity and can regulate tumor cell migration and invasion^31^, we investigated if BMPER expression can regulate cell invasion and migration in fibroblasts. Therefore, BMPER was knocked down using BMPER-specific siRNA in MRC5 cells, primary normal and IPF lung fibroblasts. Downregulation of BMPER with specific siRNA markedly reduced cell invasion (Fig. 6a,b) and migration (Fig. 6c,d). To investigate the potential mechanism of increased cell invasion and migration, we measured matrix production after knocking down BMPER. Generally, matrix facilitates migratory^38^ and invasive processes of fibrotic fibroblasts in IPF^33^. When BMPER was knocked down in fibroblasts, the HA expression was significantly diminished (Fig. 6e,f). These data indicate that BMPER regulates matrix production and fibroblast behavior.

### 5′-azacytidine attenuates lung fibrosis in mice *in vivo*

**Figure 7:**
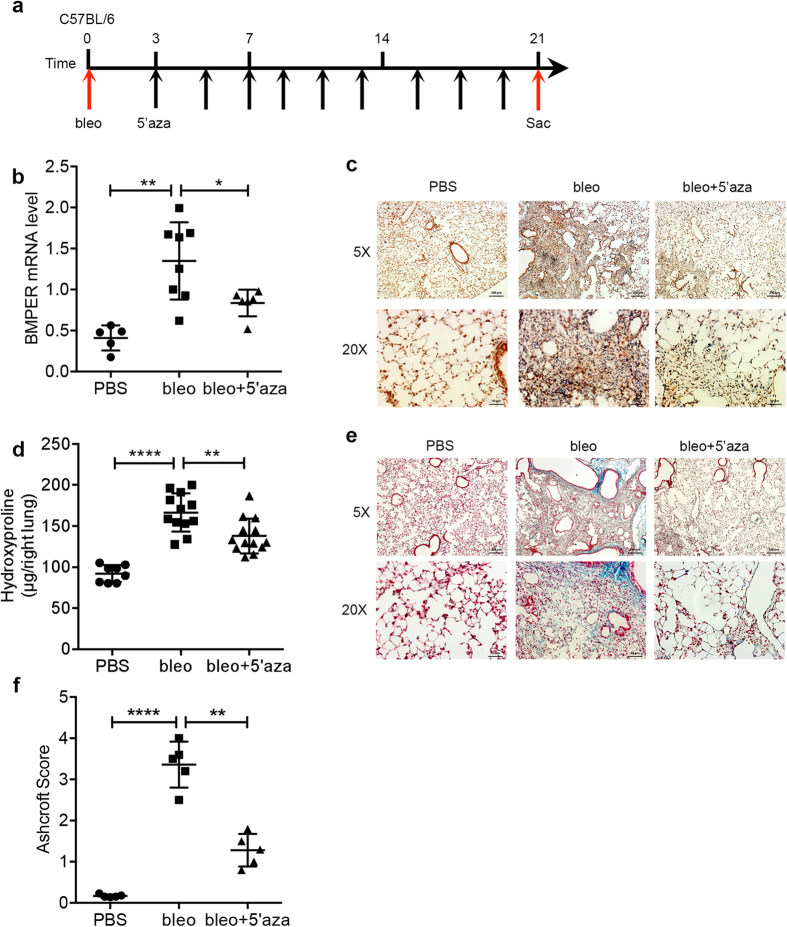
Decreased BMPER expression with 5′-azacytidine attenuated lung fibrosis in mouse *in vivo*. (**a**) Experimental strategy for *in vivo* treatments. Mice were given bleomycin (2.5 U/kg) at day 0, and followed by intraperitoneal injection of with 5′-azacytidine (1 mg/kg) every other day from day 3 to day 21. Mice were sacrificed at day 21 and lung tissues were harvested for analysis. (**b**) BMPER mRNA expression in mouse lung fibroblasts from mice treated with PBS (n = 5), bleomycin only (n = 8) or bleomycin and 5′-azacytidine (n = 6), 3 weeks after bleomycin treatment (*P < *0.05;* **P < 0.01; data are mean and SEM, one-way ANOVA analysis). (**c**) Immunohistochemical staining of BMPER in lung tissues of mice treated with PBS, bleomycin only, or bleomycin with 5′-azacytidine (scale bars, 5×, 200 μm; 20×, 50 μm). (**d**) Hydroxyproline content from mice treated with PBS (n = 8), bleomycin only (n = 12) or bleomycin and 5′-azacytidine (n = 13) (**P < 0.01; ****P < 0.0001; data are mean and SEM, one-way ANOVA analysis). (**e**) Thichrome staining of lungs from mice treated with PBS, bleomycin only, or bleomycin and 5′-azacytidine (scale bars, 5×, 200 μm; 20×, 50 μm). (**f**) Ashcroft score from mice treated with PBS (n = 5), bleomycin only (n = 5) or bleomycin and 5′-azacytidine (n = 5), 3 weeks after bleomycin treatment (**P < 0.01; ****P < 0.0001; data are mean and SEM, one-way ANOVA analysis. bleo, bleomycin; 5′aza, 5′azacytidine; Sac, sacrifice).

Given our *in vitro* results that BMPER expression was regulated by 5′-azacytidine at the transcriptional level in human lung fibroblasts, we treated C57BL/6 mice with bleomycin either with or without the demethylation agent 5′-azacytidine (Fig. 7a). Please note that we used 1 mg/kg 5′-azacytidine intraperitoneally, and the dose was much lower compared with the dose of 10 mg/kg used in the Bechtel’s study^11^. We observed a significant increase of BMPER in the lung fibroblasts of mice that received bleomycin-treated compared to that of PBS controls. BMPER expression was decreased in the lung fibroblasts of the mice received additional 5′-azacytidine treatment compared to bleomycin only, the expression of BMPER was significantly different (Fig. 7b,c). Similar to human BMPER regulation, mouse BMPER expression was also regulated by 5′-azacytidine at transcriptional level (Fig. S5). Collagen accumulation was significantly reduced in 5′-azacytidine treated lung tissue as determined by hydroxyproline assay (Fig. 7d) and Masson’s trichrome staining (Fig. 7e). Lung fibrosis was attenuated as assessed with histopathological Ashcroft score (Fig. 7f). Taken together, demethylation with 5′-azacytidine regulates BMPER expression and reduces lung fibrosis in mice *in vivo*.

## Discussion

Our studies demonstrated the profibrotic role of BMPER in pulmonary fibrosis. First, BMPER mRNA and protein levels were highly expressed in IPF lung fibroblasts compared to normal lung fibroblasts. Second, down-regulation of BMPER caused a decrease in cell invasion and migration capacities in IPF lung fibroblasts. Third, BMPER expression correlated with the extracellular matrix production, since reduced expression of BMPER, either through demethylation or siRNA inhibition, was accompanied by a decrease of the matrix production. Our previous study showed that cell invasion is increased in IPF fibroblasts^33^. Moreover, IPF lung fibroblasts have an increased migratory capacity compared to normal lung fibroblasts^4^,^38^. But the mechanism of fibroblast activation in IPF is not completely clear. We showed here that BMPER in IPF lung fibroblasts might be responsible for the activation of fibrotic fibroblasts. At the molecular level, we showed that BMPER regulates fibrosis through mediating TGF-β/BMP signaling pathway, in which BMPER may activate TGF-β signaling and inhibit BMP signaling pathway. Both TGF-β and BMP4 have been shown to play roles in regulating tissue fibrosis^18^. For example, TGF-β is a key cytokine in the pathogenesis of IPF and its signaling components have been shown to play a pivotal role in regulating tissue fibrosis^15^,^39^,^40^. And in bleomycin-induced lung fibrosis, TGF-β and BMP signaling followed an inverse course, with dynamic activation of TGF-β signaling and repression of BMP signaling activity. Modulating the balance between BMP and TGF-β may be a therapeutic target in fibrotic lung disease^41^.

Epigenetic regulation has been shown to play a role in fibrogenesis^42^. We previously showed non-coding RNAs play a role in lung injury and fibrosis^43^,^44^. In the kidney, RASAL1 was reportedly associated with the perpetuation of fibroblast activation and fibrogenesis^11^. In the lung, altered DNA methylation of Thy-1, prostaglandin E receptor 2, chemokine CXCL10 (IP-10), p14^ARF^ and α-SMA has been shown in IPF and bleomycin-induced lung fibrosis^7^,^8^,^39^,^40^,^45^,^46^. Extensive DNA methylation changed in CpG islands in IPF lungs have been reported^6^,^9^. 5′-azacytidine which is a potent inhibitor of DNA methylation has been shown to have two distinct mechanisms: cytotoxicity and demethylation^12^. Treatment with 5′-azacytidine in mice *in vivo* has been shown a global demethylation event^11^. In this study, much lower dose of 5′-azacytidine was used to minimize the cytotoxicity and retain its ability as a potent inhibitor of DNA methylation.

We further demonstrated that the demethylation agent 5′-azacytidine regulated BMPER expression through the transcriptional level *in vitro* and attenuated lung fibrosis in mice *in vivo*. It is consistent with previous reports that epigenetic mechanisms control gene expression and are likely to regulate the IPF transcriptome^47^. *In vivo* experiment, we saw that global demethylation with 5′-azacytidine decreased the expression of BMPER and extracellular matrix, but did not completely abrogate lung fibrosis following bleomycin-induced lung fibrosis in mice. We tried high-dose (10 mg/kg) of 5′-azacytidine in mouse models *in vivo* as in a previous report^11^ and it caused severe cytotoxic effect in our experimental conditions. Thus, the use of low-dose may take advantage of the demethylating effects of the drug in the current study, allowing more frequent or long-term administrations while minimizing cytotoxic side effects. For the demethylating agents currently being used in mice in lung fibrosis models, the important issue that must be carefully evaluated is the ideal dose of the 5′-azacytidine and the efficiency of the drug.

However, we also recognized that there are a few limitations with the current investigation. Although we showed that 5′-azacytidine regulated BMPER promoter activity, more detailed analysis such as the promoter sequence requirement, the possible methylation/demethylation enzymes mediated methylation, and the methylation of BMPER promoter in IPF fibroblasts are needed. We showed that BMPER is upregulated in IPF lung fibroblasts, however the role of BMPER in alveolar type II cells was not investigated. The regulation of BMPER, and the role of BMPER in mediating TGF-β/BMP signaling may differ in alveolar type II cells compared to lung fibroblasts. We recently showed that Fstl1 promoted TGF-β signaling and inhibits BMP signaling in epithelial cells, whereas in fibroblasts Fstl1 promotes TGF-β signaling without altering BMP signaling^13^. Additional studies are required to gain the mechanisms that cause BMPER promoter be methylated and whether BMPER promoter only is sufficient to induce lung fibrosis. These questions are currently being studied in our laboratory.

Therefore, further investigation into the mechanisms under which BMPER regulates lung fibrosis could provide a novel target for the development of therapeutics for patients with pulmonary fibrosis.

## Materials and Methods

### Fibroblast isolation and culture

Human lung fibroblasts were isolated from surgical lung biopsies or lung transplant explants obtained from patients with IPF and healthy donors as previously reported^48^. And mouse lung fibroblasts were obtained as previously reported^13^. Cells from passage 4 to 7 were used. Human lung fibroblast cell line (MRC5) and human embryonic kidney cell line (HEK293) were obtained from American Type Culture Collection (ATCC, Manassas, VA, USA). All experiments were approved by the Cedars-Sinai Medical Center Institutional Review Board (CR00008648) and in accordance with the guidelines outlined by the board.

### Western blotting

The expression of BMPER, Collagen1 (Col1), Phospho-Smad1 (p-Smad1), Smad1, Phospho-Smad3 (p-Smad3) and Smad3 were evaluated by western blotting essentially as described^33^. For western blotting analysis, the following antibodies were used: BMPER (ab75183, 1:1000, Abcam, Cambridge, MA, USA), Collagen1 (ab21285, 1:250, Abcam), p-Smad1 (5753s, 1:1000, Cell Signaling, Danvers, MA, USA), Smad1 (9743s, 1:1000, Cell Signaling), p-Smad3 (9520p, 1:1000, Cell Signaling), Smad3 (9523s, 1:1000, Cell Signaling) and β-actin (12620, 1:1000, Cell Signaling). The secondary antibodies used were Peroxidase AffiniPure F(ab’)2 Fragment Donkey Anti-Rabbit IgG (H+L) (Code: 711-036-152, 1:5000, Jackson ImmunoResearch, West Grove, PA, USA) and Peroxidase AffiniPure Goat Anti-Mouse IgG, Fcγ Subclass 1 Specific, (Code: 115-035-205, 1:5000, Jackson ImmunoResearch,). Pierce ECL Western Blotting Substrate (Cat. # 32106, Life Technologies, Grand Island, NY, USA) was used to visualize on a ChemiDoc MP Imaging System (Bio-RAD, Philadelphia, PA, USA). Quantitative densitometric analysis relative to β-actin was used to aid clarity.

### Quantification of mRNA

Total RNA was purified from lung fibroblasts after various treatments using RNeasy mini kit (Qiagen, Valencia, CA, USA). The quality of RNA was verified.

Real-time RT-PCR was used to quantify the relative mRNA levels in human and mouse lung fibroblasts using gene-specific primers. The relative expression levels of the gene were determined against GAPDH levels in the samples. The following primers were used: human HAS2 (GenBank accession no. NM_005328) forward, 5′-TCGCAACACGTAACGCAAT-3′; human HAS2 reverse, 5′-ACTTCTCTTTTTCCACCCCATTT-3; human GAPDH (GenBank accession no. NM_002046) forward, 5′-CCCATGTTCGTCATGGGTGT-3′; human GAPDH reverse, 5′-TGGTCATGAGTCCTTCCACGATA-3; human BMPER (GenBank accession no. NM_133468) forward, 5′-CTGCACAGCTTGTACCTGCA-3′, human BMPER reverse 5′-TTGCAAACCACAGTAGAGTC-3′; human COL1 (GenBank accession no. NM_000088) forward, 5′-GTGTTGTGCGATGACG-3′, human COL1 reverse, 5′-TCGGTGGGTGACTCTG-3′; Mouse BMPER (GenBank accession no. NM_028472) forward, 5′-GGTGCGCTGTGTTGTTCATT-3′, mouse BMPER reverse, 5′-TTCTCTCACGCACTGTGTCC-3′; mouse GAPDH (GenBank accession no. NM_001289726) forward, 5′-ATCATCTCCGCCCCTTCTG-3′, mouse GAPDH reverse, 5′-GGTCATGAGCCCTTCCACAAC-3′.

### Histology and immunohistochemistry

Mice were sacrificed at various time points after bleomycin treatment under anesthesia. The trachea was cannulated, and the left lungs were inflated with 0.5 mL of 10% neutral buffered formalin. Mouse lung tissue was fixed, embedded in paraffin, sectioned to 5 μm slices for Masson’s trichrome^13^,^49^ and BMPER staining. Anti-BMPER (ab75183, 1:200, Abcam) and anti-rabbit HRP-DAB cell and tissue staining kit were used according to the manufacturer’s instructions (R&D systems, Minneapolis, MN, USA). The semiquantitative Ashcroft score was used to score pulmonary fibrosis^50^. In short, upon 200× magnification, each successive field was given a score ranging from 0 (normal) to 8 (total fibrous obliteration of the field). All scores from 5 sections were averaged^41^.

Paraffin-embedded sections of normal and IPF lungs were used for immunofluorescence staining. Antibodies specific for BMPER (ab75183, 1:200, Abcam) and α-SMA (F3777, 1:250, Sigma, St. Louis, MO, USA) were used for staining. Alexa Fluor-546-conjugated secondary antibody was from Life Technologies. The nucleus were labeled with DAPI (Vector lab, Burlingame, CA, USA), photographed with a Leica TCS SP5 confocal microscope, and analyzed with Leica confocal software.

### Small Interference RNA Transfection

Pre-designed siRNA against human BMPER (Cat. # AM16708, 127801, Life Technologies) and Negative Control siRNA (Cat. # 1022076, Qiangen) were used. MRC-5 cells, primary human normal and IPF lung fibroblasts growing at 50–60% confluence in the complete medium in 6-well plates were transfected with 12.5–50 pmol of either BMPER siRNA or control siRNA using the Lipofectamine RNAiMAX transfection reagent (Cat. # 13778, Life Technologies) according to the manufacture’s instructions. Cell culture supernatants and proteins were harvested 48 hours after transfection.

### Plasmid transfection

BMPER expression plasmid was purchased from Genecoppiea (EX-T6437-M61) in which BMPER cDNA (NM_133468.4) was under control of a CMV promoter. One day before transfection, MRC-5 cells, primary human normal and IPF lung fibroblasts (2.5 × 10^5^ per well) were plated in 6-well plates in 2.5 mL growth medium without antibiotics. The cells were transfected with 2–4 μg BMPER plasmid and control plasmid using Lipofectamine 2000 (Cat. # 11668, Life Technologies) according to the manufacture’s instructions. Six hours after transfection, the transfectants were changed to the growth medium and 48 hours later, cells were harvested for proteins.

### Demethylation treatment

5′-azacytidine (A2385, Sigma) was dissolved in serum free DMEM and given to mice at 1 mg/kg intraperitoneally from day 3 after bleomycin treatment (2.5 U/kg) every other day until day 21. Mice were anesthetized and lungs were harvested for RNA preparation, protein isolation, or fibroblast isolation on day 21.5 μM of 5′-azacytidine was used for cell culture.

### Matrigel invasion assay

The characterization of the invasive behavior of fibroblasts isolated from IPF lungs was performed as described previously^33^,^51^. Equal numbers of fibroblasts (0.5 × 10^5^ cells) were plated into the top BioCoat Matrigel Invasion Chamber (BD Biosciences), and DMEM containing 10 ng/mL PDGF (R&D, Minneapolis, MN, USA) was loaded into the bottom chamber. After 24 hours incubation at 37 °C with 5% CO_2_, the polycarbonate filters with the invaded cells were fixed and stained with the Protocol Hema 3 stain set (Fisher Healthcare, Pittsburgh, PA, USA). Matrigel matrix and non-invading cells on the upper surface of the filter were removed by wiping with a cotton swab, and the filters were removed from the insert using a scalpel blade and were mounted onto glass slides. The invading cells of each sample were counted in five randomly selected fields under a microscope at 400× magnification. The data from 5 fields were averaged. To ensure consistency, each sample from a specific patient has performed in triplicate filters. The average of triplicates was determined for each patient.

### Fibroblast migration

Modified Boyden chambers containing 8 μm pores (24-well Transwell plate, Sigma) were used to measure the chemotaxis of lung fibroblasts to PDGF as previously described^32^. The chambers were coated with 10 μg/ml fibronectin (R&D) in PBS for 2 hours at 37 °C with 5% CO_2_. Primary lung fibroblasts (5 × 10^4^ cells) diluted in serum-free DMEM containing 1% BSA were loaded into the top chamber, and 500 μL of serum-free DMEM containing 10 ng/ml PDGF was loaded into the bottom chamber. After 4 hours incubation at 37 °C with 5% CO_2_, the fibroblasts that migrated across the fibronectin-coated filter were stained with Protocol Hema3 stain set and non-migrating cells on the upper surface of the filter were removed by wiping with a cotton swab, and the filters were removed from the insert using a scalpel blade and were mounted onto glass slides. The cells of each sample were counted in five randomly selected fields under a microscope at 400× magnification. The data from 5 fields were averaged. To ensure consistency, each sample from a specific patient has performed in triplicate filters. The average of triplicates was determined for each patient.

### Promoter constructs and luciferase assay

The BMPER promoter from −465 to +1 was amplified by PCR from mouse and human genomic DNA with the following primers: mouse (−465 to +1) forward: 5′-ATCCCTCGAGCCCGCGAGTGAA-3′, mouse (−465 to +1) reverse: 5′-CGACAAGCTTGGCGCACGCGAA-3′; human (−465 to +1): forward: 5′-AGCTCTCGAGATGAGCGGTGGG-3′, human (−465 to +1): reverse: 5′-CGAGAAGCTTGCGCGTCTCCAC-3′. The amplified product was inserted into the HindIII/XhoI sites of the luciferase expression vector PGL3 immediately downstream of the luciferase gene.

HEK 293 cells were transfected with the BMPER promoter luciferase report vectors and empty vector, as well as Renilla-luciferase as an internal control by using Lipofectamine 2000 according to the manufacture’s protocol. Six hours after transfection, the transfectants were changed to DMEM + 2% FBS medium overnight. Then the cells were treated with 5′-azacytidine at 5 μM for 24 hours. For luciferase assay, Firefly and Renilla luciferase activities were measured consecutively by using dual luciferase assays (Promega, Madison, WI, USA) 48 hours after transfection according to manufacture’s suggestions.

### Enzyme-linked immunosorbent assay (ELISA)

The concentration of HA was determined by HA ELISA-like assay (Hyaluronan DuoSet ELISA kit, DY3614, R&D Systems) following the manufacturer’s instructions.

### Assessment of cell viability

Cell viability was evaluated using a colorimetric assay with 3-[4,5-dimethylthiazol-2-yl]-2,5-diphenyltetrazolium bromide (MTT). Human lung fibroblasts were seeded into a 24-well plate and starved with DMEM containing 2% FBS for 24 hours. The cells were then treated with 5′-azacytidine at 5 μM for additional 24 hours. The MTT staining was performed and the optical density of each well was measured at 595 nm. The OD of control wells was taken as 100% cell viability.

### Bleomycin lung injury and fibrosis model

All experiments were carried out using 8–12 weeks old C57BL/6 female mice (The Jackson Laboratory, Bar Harbor, Maine, USA). Bleomycin (bleo) (Hospira, San Jose, CA, USA) was injected intratracheally at a dose of 2.5 U/kg body weight as described previously^33^,^49^. At designated time points after bleomycin injection, mice were anesthetized by ketamine (Vedco) and xylazine (Anased, Shenandoah, IA, USA) injection, and lungs were harvested for RNA preparation, protein isolation, or fibroblast isolation. All mouse experiments were approved by IACUC at Duke University (A134-12-05) and Cedars-Sinai Medical Center (004751).

### Hydroxyproline assay

Collagen content in the right lung tissue was measured with the conventional hydroxyproline method^33^.

### Affymetrix cDNA microarray

Cultured fibroblasts (at passage 4) from 3 IPF patients were treated with 5′-azacytidine 5 μM for 24 hours. RNA were extracted and hybridized on Affymetrix microarrays. Differentially expressed genes between groups (IFP treated vs non-treated) selected with following cutoff *P* <= 0.05 and fold change >= 1.5. The array data have been submitted to the National Center for Biotechnology Information Gene Expression Omnibus database (accession no: GSE69764).

### Statistical analysis

Data are presented as means ± SEM. We assessed differences in measured variables using the paired or unpaired two-sided Student t test. Data among groups were compared by One-way ANOVA with Tukey post test. Statistical difference was accepted at *P* < 0.05. Prism version 5.0 (GraphPad) was used to generate graphs and statistical analyses.

## Additional Information

**How to cite this article**: Huan, C. *et al.* Methylation-mediated BMPER expression in fibroblast activation *in vitro* and lung fibrosis in mice *in vivo*. *Sci. Rep.* **5**, 14910; doi: 10.1038/srep14910 (2015).

## Supplementary Material

Supplementary Information
