# Supplementary material for: Methylation-mediated BMPER expression in fibroblast activation in vitro and lung fibrosis in mice in vivo
Source: Sci Rep. 2015 Oct 7;5:14910. doi: 10.1038/srep14910 (PMC4595647; doi:10.1038/srep14910)
Supplement: Supplementary Information [file srep14910-s1.doc]

**Supplementary Information**

**Methylation-mediated BMPER expression in fibroblast activation in vitro and lung fibrosis in mice in vivo**

Caijuan Huan^1, 2^, Ting Yang^1^, Jiurong Liang^2^, Ting Xie^2^, Luis Cheng^2^, Ningshan Liu^2^, Adrianne Kurkciyan^2^, Jessica Monterrosa Mena^2^, Chen Wang^3^, Huaping Dai^1^, Paul W. Noble^2^, and Dianhua Jiang^2*^

Supplementary Figure S1

Supplementary Figure S2

Supplementary Figure S3

Supplementary Figure S4

Supplementary Figure S5


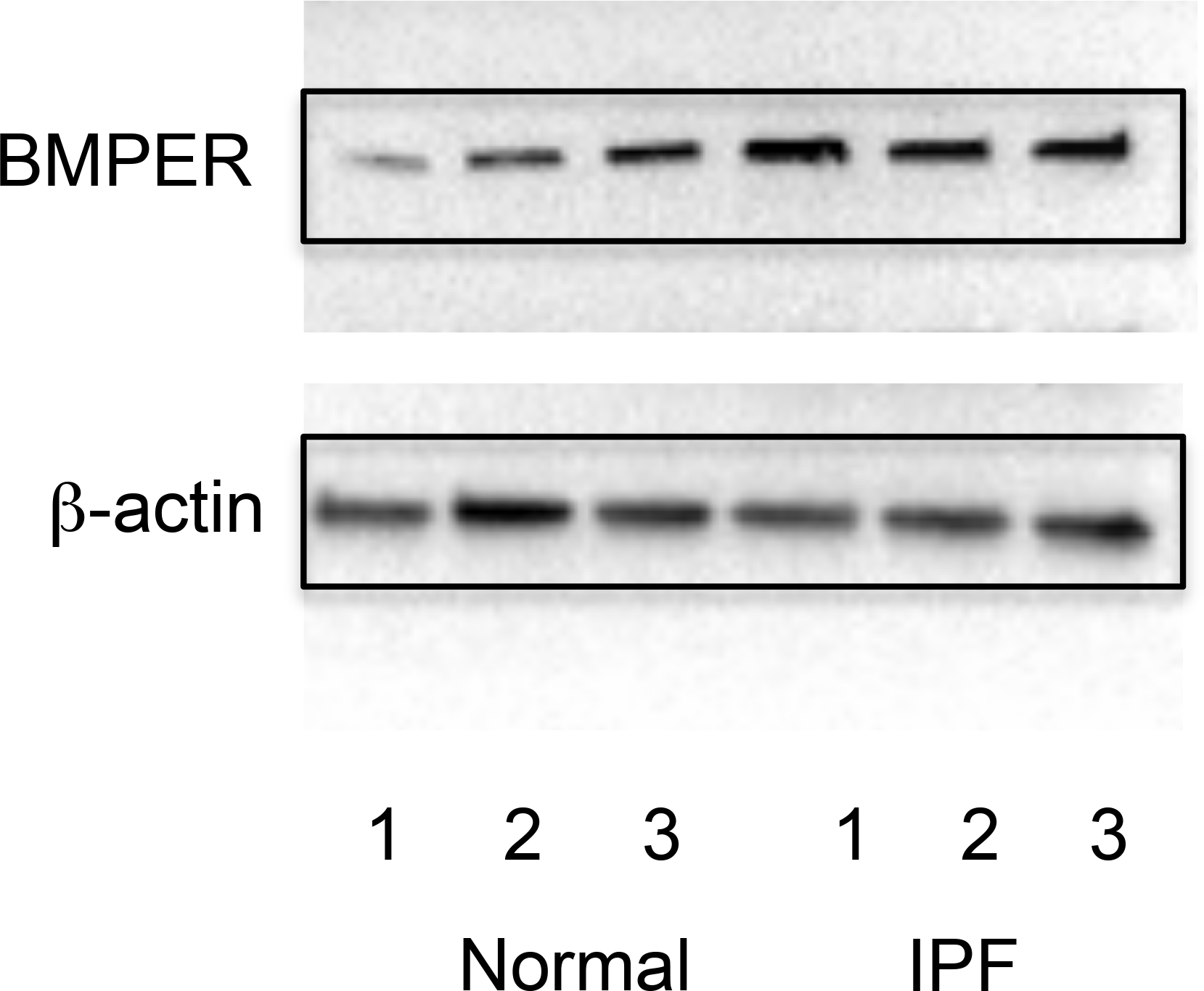


**Figure S1. Full-length gels and blots for key data in the main figures for Figure 1a.**


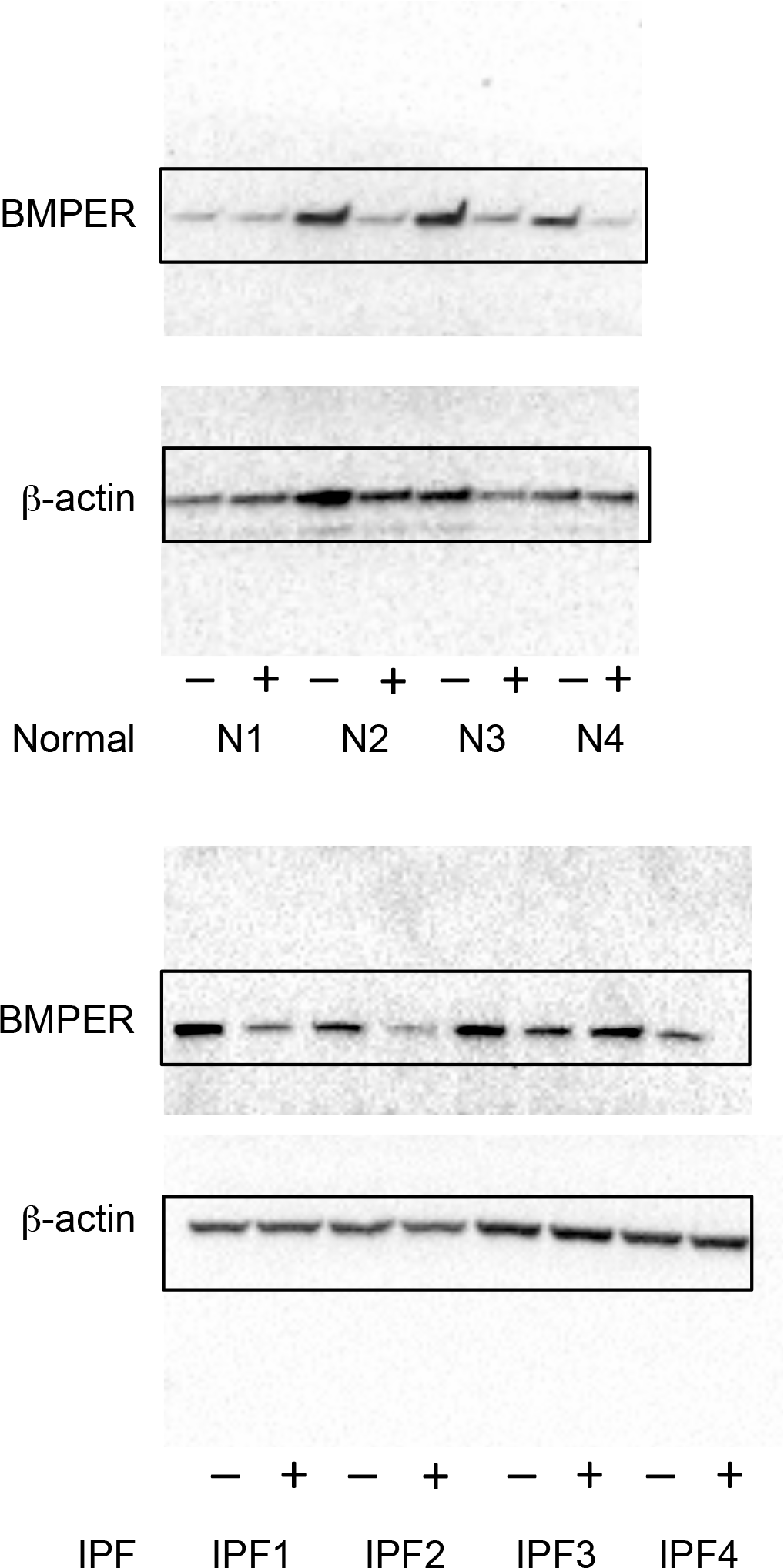


**Figure S2. Full-length gels and blots for key data in the main figures for Figure 2a and Figure 2c.**


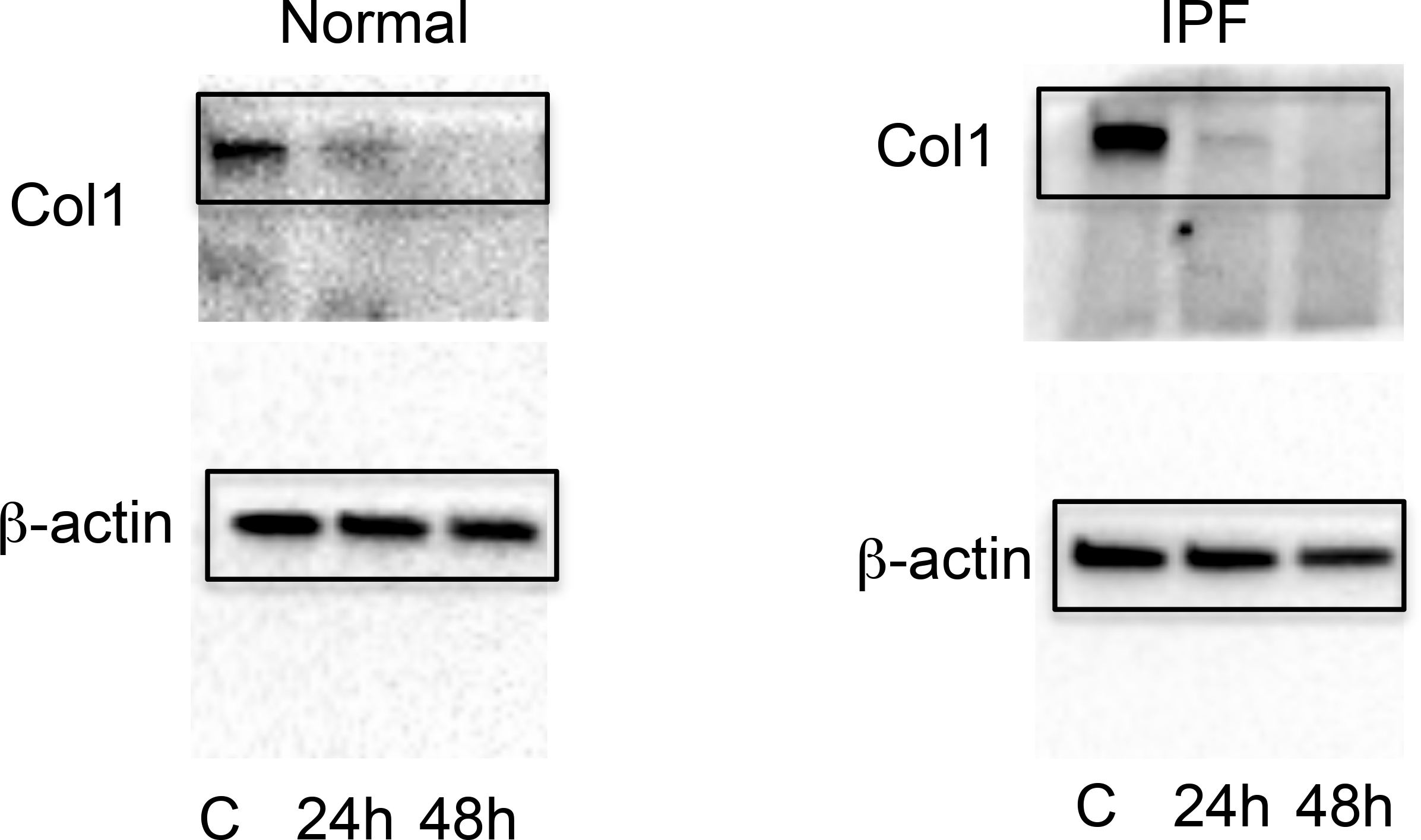


**Figure S3. Full-length gels and blots for key data in the main figures for Figure 3a and Figure 3b.**

**
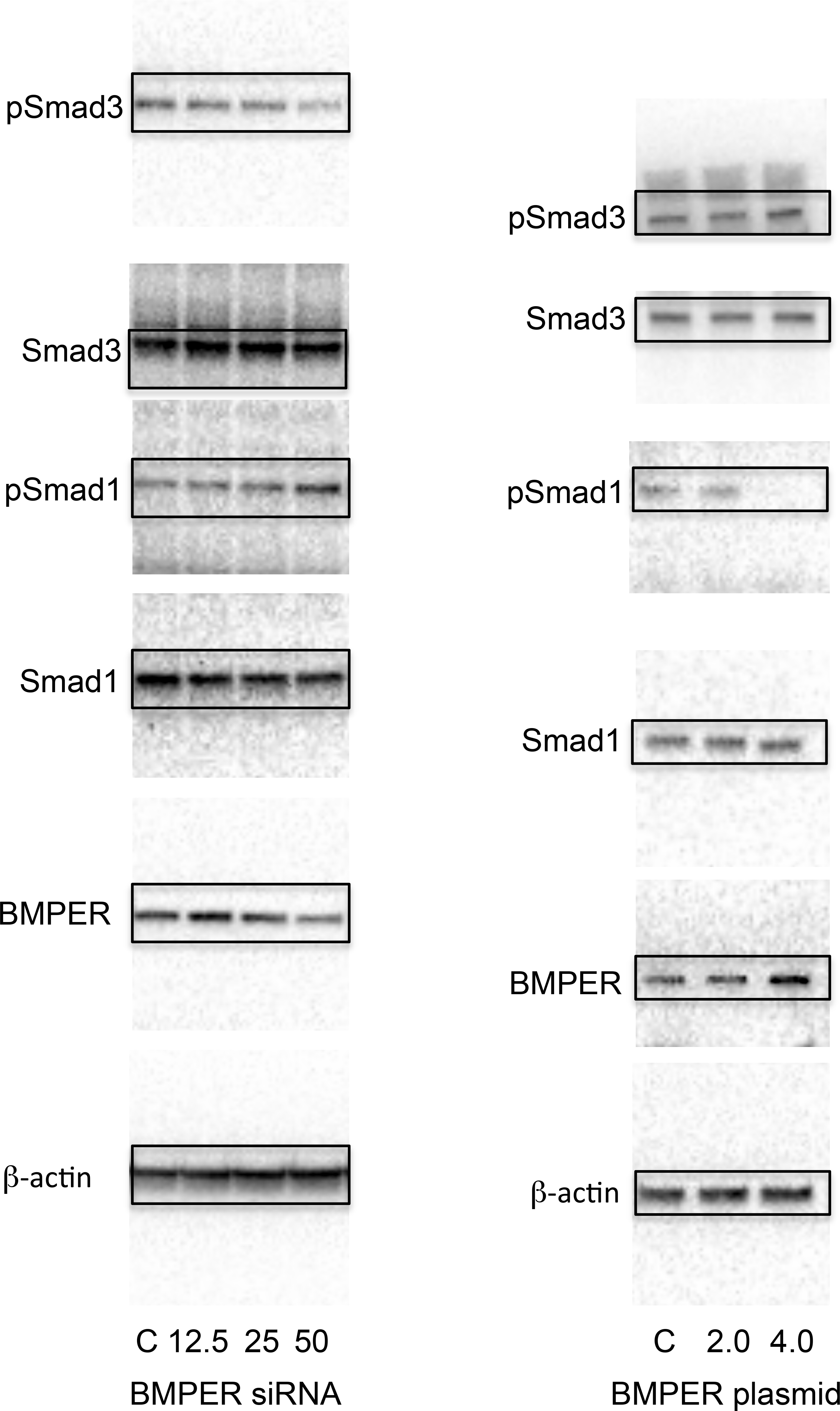
**

**Figure S4. Full-length gels and blots for key data in the main figures for Figure 5a and Figure 5b.**

**
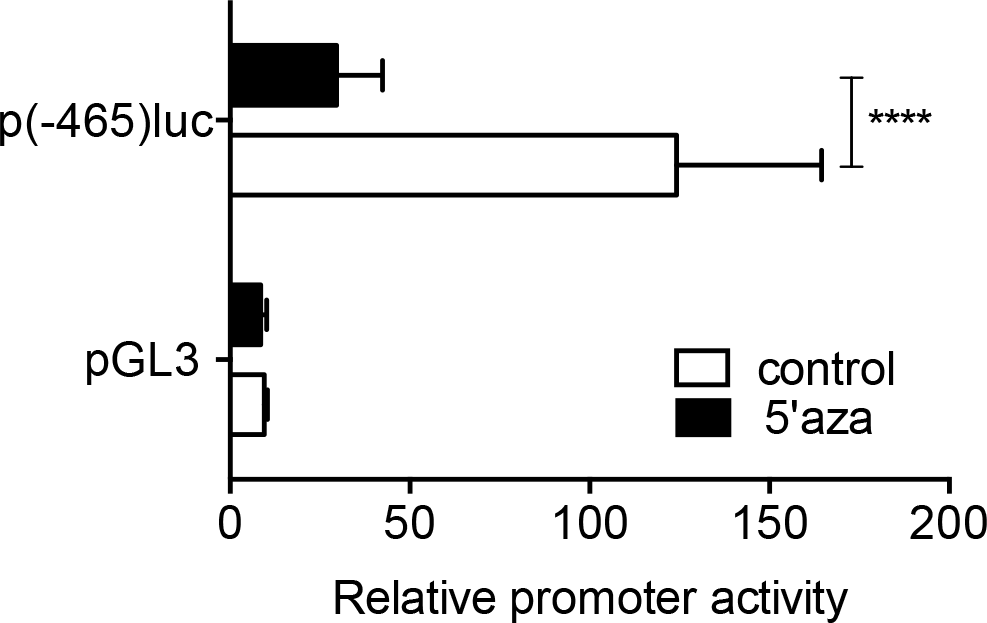
**

**Figure S5. Demethylation regulates BMPER promoter activity.**

The mouse BMPER promoter to drive luciferase (p(-465) luc) along with vector control (pGL3) were transfected into 293HEKs cells. 24 hours later, the transfectants were treated with 5’-azacytidine, and promoter activities were measured 48 hours after transfection. 5’-azacytidine treatment reduced promoter activities (****, *P* < 0.0001).
